# Microorganisms in the phyllosphere of Norway spruce controlling nitrous oxide dynamics

**DOI:** 10.1093/ismeco/ycaf196

**Published:** 2025-11-03

**Authors:** Dhiraj Paul, Inga Paasisalo, Anuliina Putkinen, Christopher M Jones, Lukas Kohl, Sara Hallin, Mari Pihlatie, Henri M P Siljanen

**Affiliations:** Department of environmental and biological sciences, University of Eastern Finland, Kuopio 70210, Finland; Department of environmental and biological sciences, University of Eastern Finland, Kuopio 70210, Finland; Environmental Soil Sciences, Department of Agricultural Sciences, University of Helsinki, Helsinki 00790, Finland; Institute of Atmospheric and Earth System Research (INAR), University of Helsinki, Helsinki 00790, Finland; Department of Microbiology, University of Helsinki, Helsinki 00790, Finland; Department of Forest Mycology and Plant Pathology, Swedish University of Agricultural Sciences Box 7026, Uppsala 750 07, Sweden; Department of environmental and biological sciences, University of Eastern Finland, Kuopio 70210, Finland; Department of Forest Mycology and Plant Pathology, Swedish University of Agricultural Sciences Box 7026, Uppsala 750 07, Sweden; Environmental Soil Sciences, Department of Agricultural Sciences, University of Helsinki, Helsinki 00790, Finland; Institute of Atmospheric and Earth System Research (INAR), University of Helsinki, Helsinki 00790, Finland; Department of environmental and biological sciences, University of Eastern Finland, Kuopio 70210, Finland

**Keywords:** greenhouse gas (GHG), nitrous oxide, *nos*Z, phyllospheric, microbial community, probe captured metagenomics

## Abstract

Current climate change assessments and greenhouse gas flux models often lack information on the microbiological processes that consume atmospheric nitrous oxide (N_2_O), a potent greenhouse gas. There is limited understanding of phyllospheric microorganisms controlling N_2_O exchange. In this study, we determined the microbial potential for N_2_O consumption in aboveground vegetation in boreal forests. For this, we collected shoot samples from upland spruce forests in Finland and used a novel targeted metagenomics approach with a hybridization capture of gene-specific probes. Most of the samples contained *nos*Z genes, encoding the N_2_O reductase. Phylogenetic placement showed a significantly higher relative abundance (*P* < .01) of *nos*Z Clade I than *nos*Z Clade II. Bacterial members such as Comamonadaceae, *Hydrogenophaga*, and *Paracoccus*, which all harbor *nos*Z Clade I, were found in high relative abundance in the spruce shoots across the sites, suggesting they play a role in N_2_O consumption capabilities in the spruce phyllosphere. Anoxic incubations, utilizing gas chromatography for N_2_O analyses, showed potential N_2_O consumption activity across the spruce samples. The presence of *nir*K and *nir*S suggests potential for denitrification, possibly resulting in N_2_O production. Our finding provides evidence of microbial communities in spruce canopies with potential for N_2_O exchange. Given the vast coverage of boreal forests globally, understanding the role of phyllospheric microorganisms in N₂O exchange is crucial for improving the accuracy of greenhouse gas models and enhancing climate prediction reliability.

## Background

Nitrous oxide (N_2_O) is a major, long-lived greenhouse and stratospheric ozone layer-depleting gas, mainly produced by the microbial processes nitrification and denitrification [1, 2]. The only process known to reduce N_2_O to molecular nitrogen (N_2_) in the biosphere is microbial reduction by both denitrifying and non-denitrifying microorganisms [3]. Nitrous oxide reduction is catalyzed by the enzyme N_2_O reductase, which is phylogenetically divided into two clades: Clade I and Clade II, and encoded by the *nos*Z gene [4, 5]. Several studies have investigated the role of N_2_O-producing and consuming microorganisms for net N_2_O emissions from soil; less is known about aboveground, i.e. phyllospheric, compartments despite indications that plants or their associated microbiome contribute to N_2_O fluxes [6, 7]. A recent laboratory study demonstrated that the leaf microbiome in an urban greenery plant consumes N_2_O [8], and it has also been shown that canopy nitrification serves as a significant source of soil nitrate in forest ecosystems [9], indicating the important contribution of canopy communities to nitrogen cycling. The Earth's leaf surface area is around 400 million km^2^ [8], emphasizing the critical role of the phyllosphere microbial community as a vital resource and its potentially significant role in greenhouse gas exchange. Therefore, identifying the microbial processes and specific microorganisms that are implicated in both the production and consumption of N_2_O in the phyllosphere of trees is essential for advancing our understanding of the role of forest canopies in global N_2_O budgets.

The aim of this study was to determine the prevalence of microorganisms with the capacity for N_2_O exchange in boreal spruce shoots and the N_2_O reduction activity in these shoots. However, it is challenging to isolate N-cycling genes from total DNA derived from plant tissue, primarily due to the overwhelming presence of plant DNA, but also because these functional genes are significantly less abundant compared to those involved in primary metabolism in the microbial community. To confront this issue, we employed metagenomics combined with a cutting-edge probe capture technique designed to effectively identify microbial N cycling genes within the spruce shoot samples (see detailed supplementary materials and methods). The probe capture method has distinct advantages over traditional amplicon sequencing, which can introduce PCR bias, and is more effective than shotgun sequencing for studying specific microbial functions in host-microbe ecosystems dominated by DNA from the host [10, 11]. Using targeted probes to selectively sequence specific genes also allows for gene detection of rare microorganisms.

## Results and discussion

**Figure 1:**
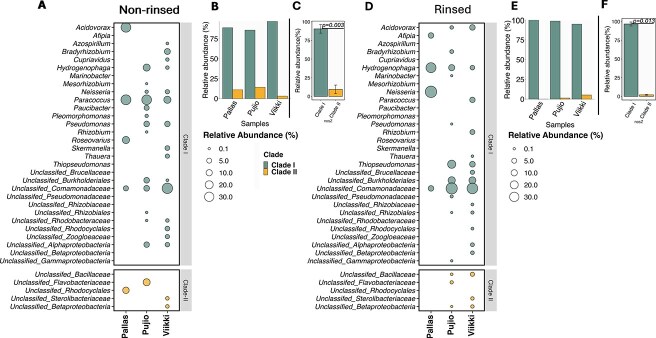
Prevalence of *nos*Z Clade I and Clade II in spruce shoots. The taxonomic distribution of *nos*Z Clade I and Clade II among the (A) non-rinsed samples (whole microbiome) and (D) rinsed samples (endophytes). The sequences that are not classified at the genus level but at the family level are designated as “unclassified” followed by the family name. The size of each circle represents the relative abundance of each taxon, while different colors (green and yellow) indicate Clade I and Clade II. Relative abundance of *nos*Z Clade I and Clade II across sites of (B) non-rinsed and (E) rinsed samples. Overall *nos*Z Clade I and Clade II distribution for (C) nonrinsed and (F) rinsed samples. All P-values are for the Wilcoxon test.

To evaluate the potential for the N_2_O exchange within the boreal forest phyllosphere, we collected samples from three locations across Finland: Pallas, Viikki, and Puijo (Details are provided in the Supplementary Material, Supplementary Fig. S1). Functional genes involved in N_2_O consumption and production were successfully identified from DNA extracted from the spruce shoots using probe-captured metagenomics (Supplementary Fig. S2). Notably, both *nos*Z clades were identified (Supplementary Fig. S3A), with *nos*Z Clade I being more abundant than Clade II in all shoot samples (Supplementary Fig. S3B). The family Comamonadaceae, along with the genera *Hydrogenophaga* and *Paracoccus*, which belong to the *nos*Z Clade I community, were present in all spruce shoot samples collected from the sites. These bacteria are previously reported as aerobic or facultatively aerobic and have the capability to reduce N_2_O [12–14], making them suitable for the phyllospheric region. The dominating *nos*Z Clade I taxa were consistently observed across both rinsed (targeting the endophytes) and non-rinsed (targeting the entire shoot microbiome) spruce shoots, with the exception of *Paracoccus* (Fig. 1A and D). *nos*Z Clade I organisms had a significantly (*P* < .01) higher relative abundance compared to *nos*Z Clade II in both rinsed and non-rinsed shoots (Fig. 1 and Supplementary  Fig.  S3). Additionally, phylogenetic analysis revealed that microorganisms belonging to *nosZ* Clade I and Clade II contain both distinct and phylogenetically comparable groups of organisms, depending on the collection site of the spruce samples (Supplementary Fig. S4).

The majority of previous research on N_2_O emission/consumption and the involvement of microorganisms in soil samples has shown either a dominance of *nos*Z Clade II or an equal abundance of the two clades [3, 15]. However, in root-associated communities, *nos*Z Clade I typically dominates over *nos*Z Clade II [9, 10]. *nos*Z Clade I is primarily found in Pseudomonadota [15], which may grow faster than organisms with *nos*Z Clade II, thereby outcompeting them [16]. Among Pseudomonadota with *nos*Z Clade I, there is a dominance of organisms with a complete denitrification pathway [17]. In addition to *nos*Z, functional genes involved in different steps of the denitrification pathway, i.e. *nir*S, *nir*K, *nor*B, *nar*G, were detected from the spruce shoot samples by captured metagenomics, both in rinsed and non-rinsed samples (Supplementary Fig. S2). This indicates that complete denitrification can occur, at least at the community level. Studies using pure cultures of denitrifying microorganisms have demonstrated that those with a complete pathway can be more competitive at low nitrate levels, as they can utilize all electron acceptors involved in the pathway [18]. Additionally, complete denitrifiers are often more metabolically flexible, allowing them to utilize a wider range of carbon substrates [19]. Altogether, this may help explain the prevalence of *nos*Z Clade I organisms in spruce shoots.

**Figure 2:**
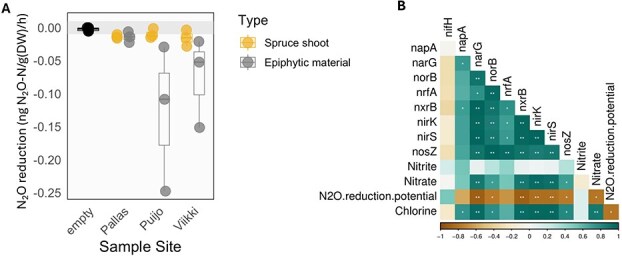
N_2_O reduction potential in spruce shoots. (A) N_2_O reduction potential measurements during incubation study of both the rinsed spruce shoots (endophytes) and rinsing media (epiphytes rinsed from the shoots, mean ± standard error, *n* = 3). The grey horizontal line indicates the zero line for the apparent N_2_O uptake. There were no significant differences between samples and control blanks at each site (Wilcoxon test). (B) Correlation analyses between nitrogen-cycling genes, tree chemical properties, and N_2_O reduction potential (^*^ = *P* < .01, ^**^ = *P* < .05; Spearman’s correlation). Relative abundances of nitrogen cycling genes were obtained from captured metagenomics and chemical data from ion estimation conducted using ion chromatography of epiphytic water samples from the incubation experiment.

To confirm the role of microorganisms in spruce shoots concerning N_2_O dynamics, we conducted an anoxic incubation study to measure the N_2_O reduction potential (detailed experimental information is provided in the supplementary methods). Our findings demonstrated that N_2_O reduction is higher in samples with epiphytic microorganisms (rinsing media) compared to those with endophytic microorganisms (rinsed spruce shoots) (Fig. 2A). There was significant variation among the individual biological samples: four out of nine showed true N_2_O reduction, distinguishing them from the controls without shoots (Fig. 2A; Supplementary Fig. S5A). These results indicate that microorganisms in the spruce phyllosphere have potential for N_2_O reduction. Additionally, we observed variation in nitrite and nitrate content in shoot surfaces (Supplementary Table S1), which could be linked to nitrogen (N) deposition from the atmosphere, explained by the proximity to a city. Furthermore, nitrite and nitrate serve as nitrogen sources for plant cells, contributing to the variation in their content. Correlation analysis also revealed a significant positive correlation (*P* < .05) between denitrification genes, including *nos*Z abundance and nitrate and nitrite content, and a negative association with total N_2_O reduction potential. This suggests that the prevalence of denitrifiers may be important for N_2_O reduction in the boreal spruce forest ecosystem (Fig. 2B).

In conclusion, our findings, derived from a novel targeted metagenomics tool, provide new insights into the phyllospheric microbes associated with spruce trees, highlighting both N_2_O production and consumption genes. Notably, we observed a higher abundance of *nos*Z Clade I among N_2_O-reducing organisms, underscoring the unique characteristics of plant-associated microbiomes. Our study centered on the reduction processes that may occur in anoxic or low-oxygen microhabitats within the phyllosphere, which can develop due to rain or dew accumulation [8]. Future studies should widen the scope to also include aerobic processes to reveal the scale of potential N_2_O production. Further, incorporating transcriptomics would advance our understanding of which N_2_O-exchanging microorganisms and processes are active under different conditions. Finally, systematic studies incorporating a diverse array of tree species across broader spatial and temporal scales are essential for accurately assessing the ecological relevance of these microorganisms and their impact on the N_2_O balance.

## Supplementary Material

FigS1_ycaf196

FigS2_ycaf196

FigS3_ycaf196

FigS4_ycaf196

FigS5_ycaf196

Supplementary_Table_1_ycaf196

## Data Availability

The data sets generated and analyzed during this study are available in the following repositories. Raw sequence data have been deposited in the NCBI SRA database under accession number SRR28227330-SRR28227352, with the Bioproject ID PRJNA1083928.

## References

[1] Inatomi M, Hajima T, Ito A. Fraction of nitrous oxide production in nitrification and its effect on total soil emission: a meta-analysis and global-scale sensitivity analysis using a process-based model. *PLoS One* 2019;14:e0219159. 10.1371/journal.pone.0219159 31291317 PMC6619742

[2] Scheer C, Fuchs K, Pelster DE. et al. Estimating global terrestrial denitrification from measured N_2_O:(N_2_O+ N_2_) product ratios. *Curr Opin Environ Sustain* 2020;47:72–80. 10.1016/j.cosust.2020.07.005

[3] Hallin S, Philippot L, Löffler FE. et al. Genomics and ecology of novel N_2_O-reducing microorganisms. *Trends Microbiol* 2018;26:43–55. 10.1016/j.tim.2017.07.003 28803698

[4] Sanford RA, Wagner DD, Wu Q. et al. Unexpected nondenitrifier nitrous oxide reductase gene diversity and abundance in soils. *Proc Natl Acad Sci USA* 2012;109:19709–14. 10.1073/pnas.1211238109 23150571 PMC3511753

[5] Jones CM, Graf DR, Bru D. et al. The unaccounted yet abundant nitrous oxide-reducing microbial community: a potential nitrous oxide sink. *ISME J* 2013b;7:417–26. 10.1038/ismej.2012.125 23151640 PMC3554408

[6] Schützenmeister K, Meurer KH, Gronwald M. et al. N_2_O emissions from plants are reduced under photosynthetic activity. *Plant Environ Interact* 2020;1:48–56. 10.1002/pei3.10015 37284131 PMC10168041

[7] Ranniku R, Schindler T, Escuer-Gatius J. et al. Tree stems are a net source of CH4 and N2O in a hemiboreal drained peatland forest during the winter period. *Environ Res Commun* 2023;5:051010. 10.1088/2515-7620/acd7c7

[8] Zhang Y, Chen Q, Yang X. et al. Unravelling the activity and presence of N_2_O reducers on urban greening tree leaves. *Plant Cell Environ* 2025;48:4770–80. 10.1111/pce.15463 40079375

[9] Guerrieri R, Cáliz J, Mattana S. et al. Substantial contribution of tree canopy nitrifiers to nitrogen fluxes in European forests. *Nat Geosci* 2024;17:130–6. 10.1038/s41561-023-01364-3

[10] Putkinen A, Siljanen HM, Laihonen A. et al. New insight to the role of microbes in the methane exchange in trees: evidence from metagenomic sequencing. *New Phytol* 2021;231:524–36. 10.1111/nph.17365 33780002

[11] Siljanen HM, Manoharan L, Hilts AS. et al. Targeted metagenomics detect a larger diversity of nitrogen and methane cycling genes in complex microbial communities than traditional metagenomics. *ISME Commun* 2025;5:1–12. 10.1093/ismeco/ycaf183 PMC12598625 41221508

[12] Bergaust L, van Spanning RJ, Frostegård Å. et al. Expression of nitrous oxide reductase in Paracoccus denitrificans is regulated by oxygen and nitric oxide through FnrP and NNR. *Microbiology* 2012;158:826–34. 10.1099/mic.0.054148-0 22174385 PMC3541799

[13] Zhou J, Ding L, Cui C. et al. High nitrite accumulation in hydrogenotrophic denitrification at low temperature: transcriptional regulation and microbial community succession. *Water Res* 2024;263:122144. 10.1016/j.watres.2024.122144 39079193

[14] Schacksen PS, Nielsen JL. Unraveling the genetic potential of nitrous oxide reduction in wastewater treatment: insights from metagenome-assembled genomes. *Appl Environ Microbiol* 2024;90:e02177–23. 10.1128/aem.02177-23 39136491 PMC11409646

[15] Jones CM, Graf DR, Bru D. et al. The unaccounted yet abundant nitrous oxide-reducing microbial community: a potential nitrous oxide sink. *ISME J* 2013a;7:417–26. 10.1038/ismej.2012.125 23151640 PMC3554408

[16] Conthe M, Wittorf L, Kuenen JG. et al. Growth yield and selection of *nos*Z clade II types in a continuous enrichment culture of N_2_O respiring bacteria. *Environ Microbiol Rep* 2018;10:239–44. 10.1111/1758-2229.12630 29457693

[17] Graf DR, Jones CM, Hallin S. Intergenomic comparisons highlight modularity of the denitrification pathway and underpin the importance of community structure for N_2_O emissions. *PLoS One* 2014;9:e114118. 10.1371/journal.pone.0114118 25436772 PMC4250227

[18] Felgate H, Giannopoulos G, Sullivan MJ. et al. The impact of copper, nitrate and carbon status on the emission of nitrous oxide by two species of bacteria with biochemically distinct denitrification pathways. Environ Microbiol 2012;14:1788–800. 10.1111/j.1462-2920.2012.02789.x 22642644

[19] Pold G, Saghai A, Jones CM. et al. Denitrification is a community trait with partial pathways dominating across microbial genomes and biomes. Nat. Commun 16:1–16. 41152293 10.1038/s41467-025-65319-5 PMC12569097

