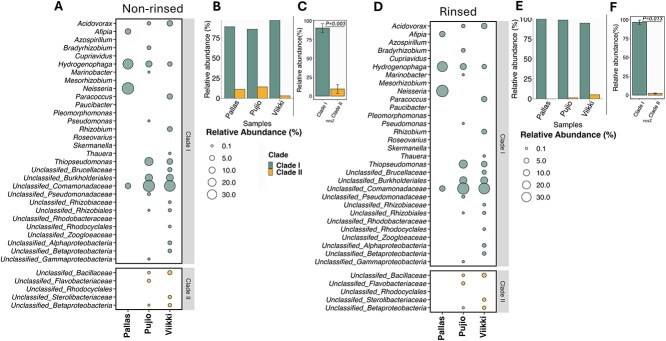# Correction to: Microorganisms in the phyllosphere of Norway spruce controlling nitrous oxide dynamic

**DOI:** 10.1093/ismeco/ycag103

**Published:** 2026-05-06

This is a correction to: Dhiraj Paul, Inga Paasisalo, Anuliina Putkinen, Christopher M Jones, Lukas Kohl, Sara Hallin, Mari Pihlatie, Henri M P Siljanen, Microorganisms in the phyllosphere of Norway spruce controlling nitrous oxide dynamics, *ISME Communications*, Volume 5, Issue 1, January 2025, ycaf196, https://doi.org/10.1093/ismeco/ycaf196

In the originally published version of the paper there was an error in Figure 1. Panels A and D were duplicated. These, and the caption to the figure, are now corrected.


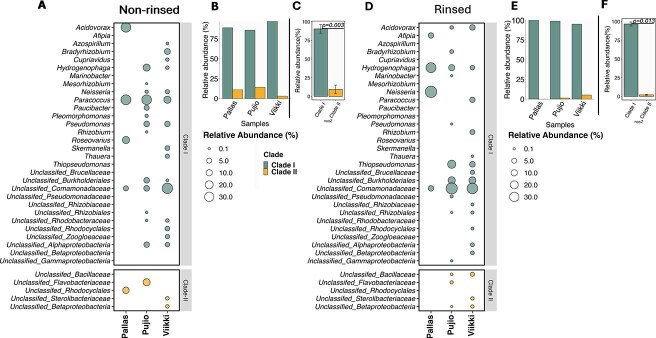


instead of: