# Supplementary figures and images for: Microorganisms in the phyllosphere of Norway spruce controlling nitrous oxide dynamics
Source: ISME Commun. 2025 Nov 3;5(1):ycaf196. doi: 10.1093/ismeco/ycaf196 (PMC12642870; doi:10.1093/ismeco/ycaf196)

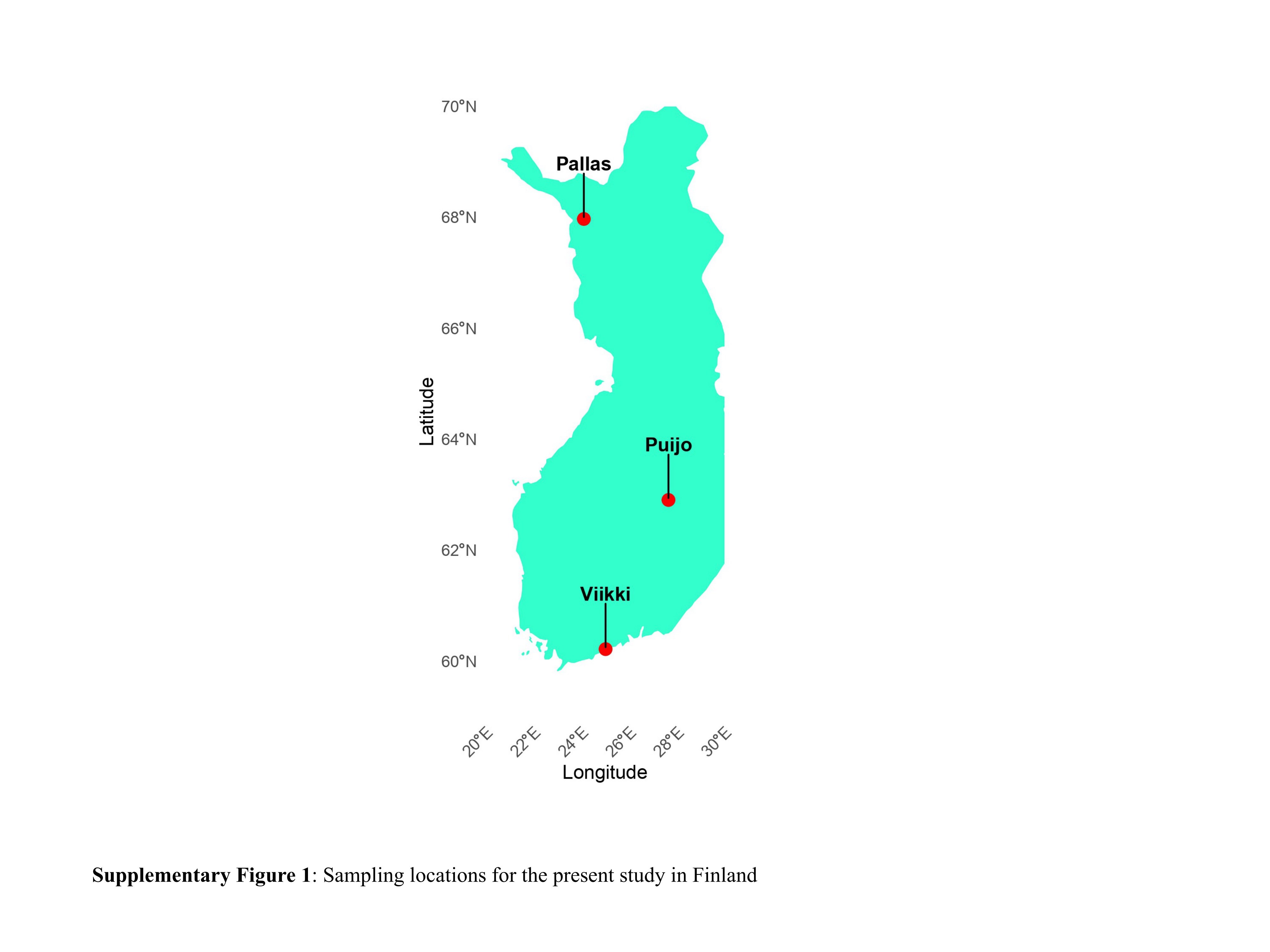

Supplement: FigS1_ycaf196 [file figs1_ycaf196.jpeg]

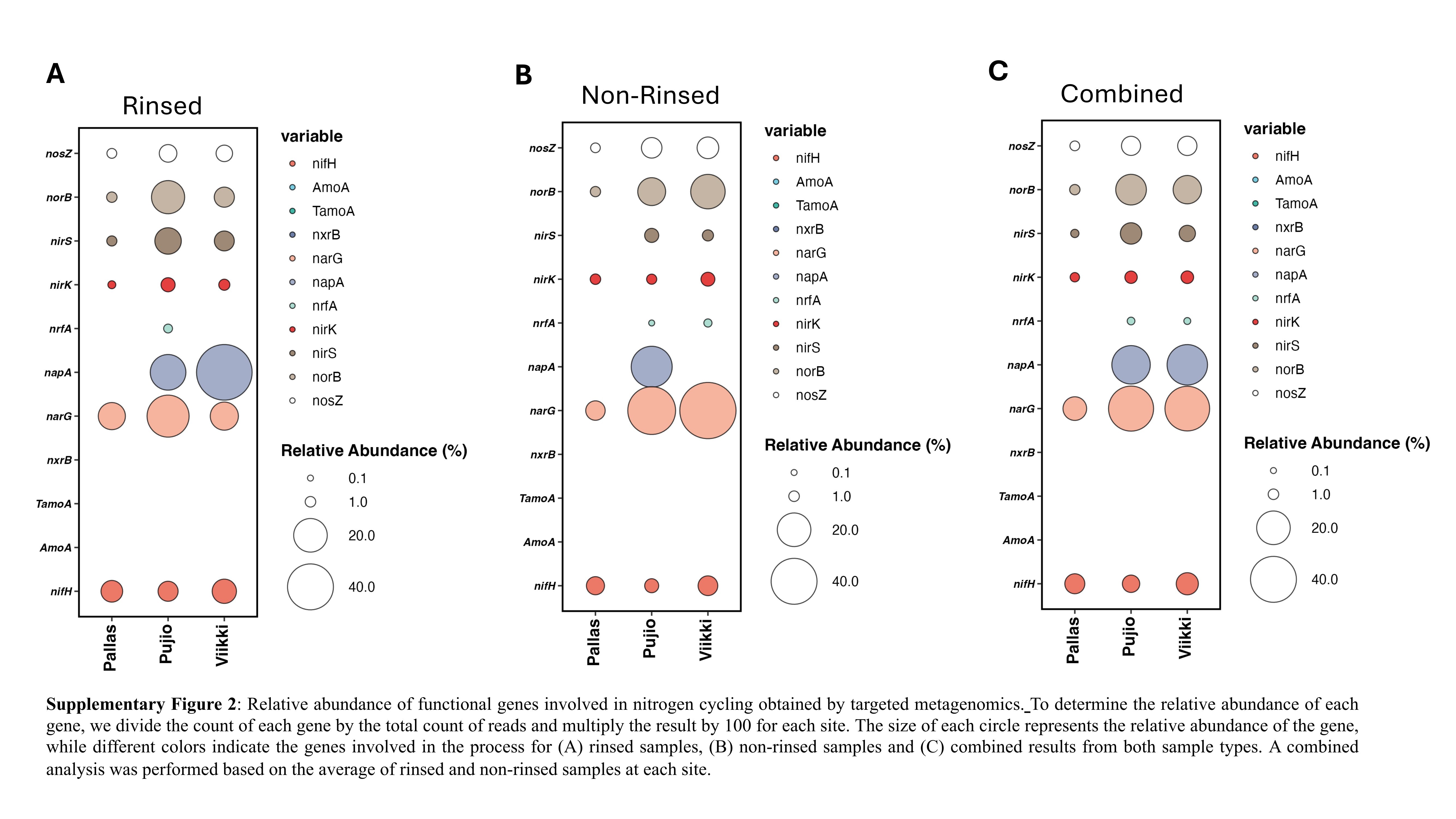

Supplement: FigS2_ycaf196 [file figs2_ycaf196.jpeg]

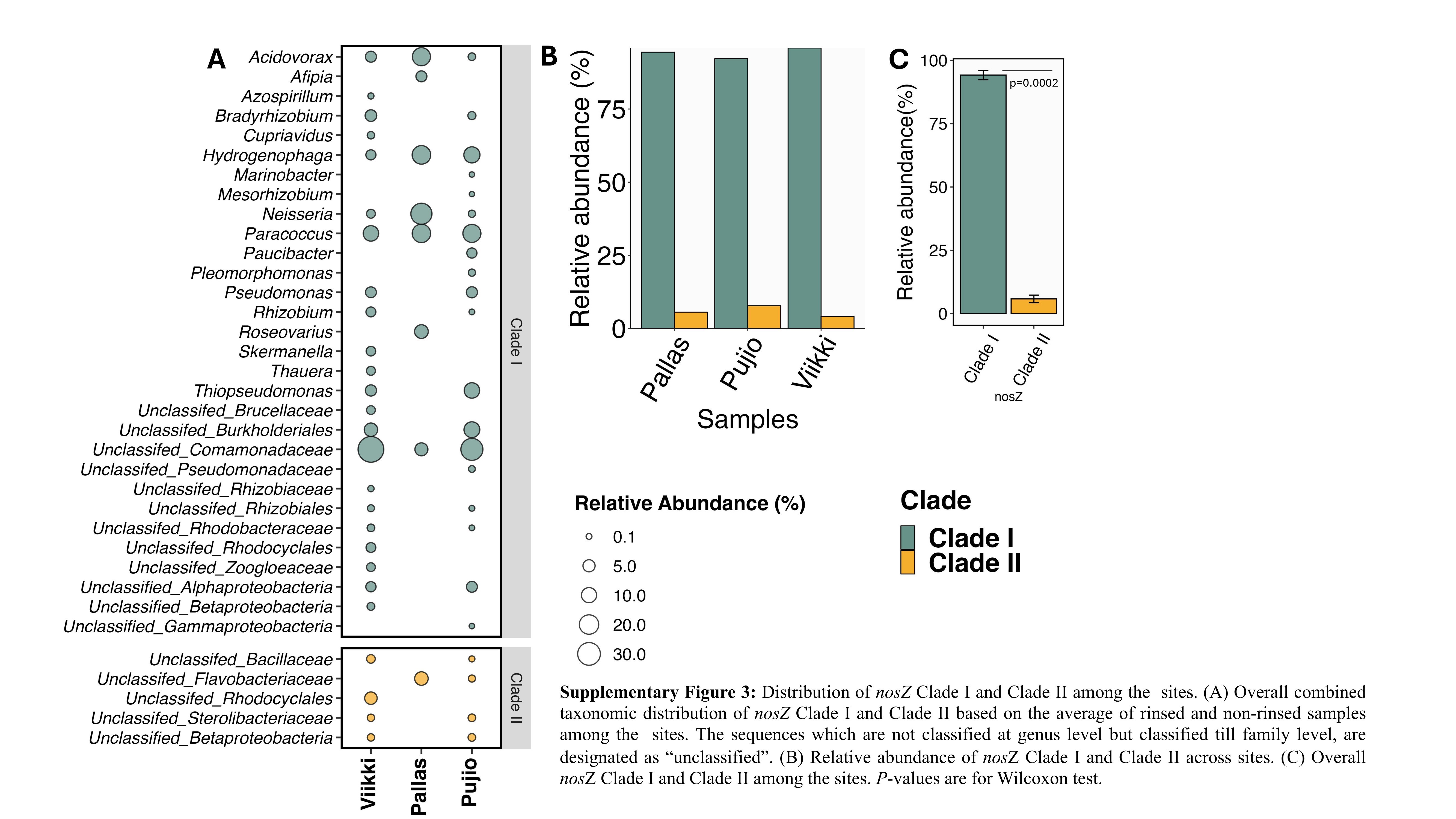

Supplement: FigS3_ycaf196 [file figs3_ycaf196.jpeg]

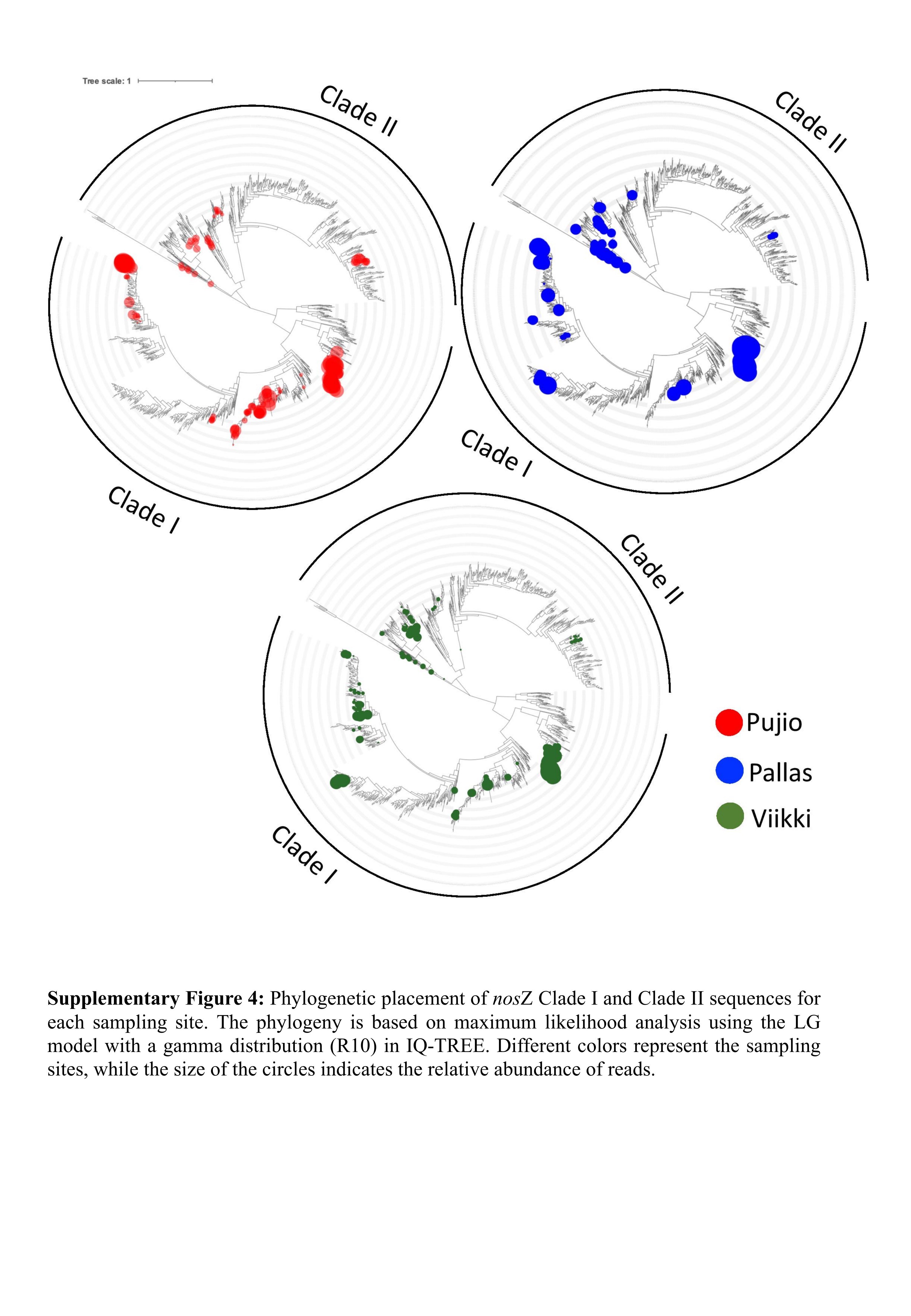

Supplement: FigS4_ycaf196 [file figs4_ycaf196.jpeg]

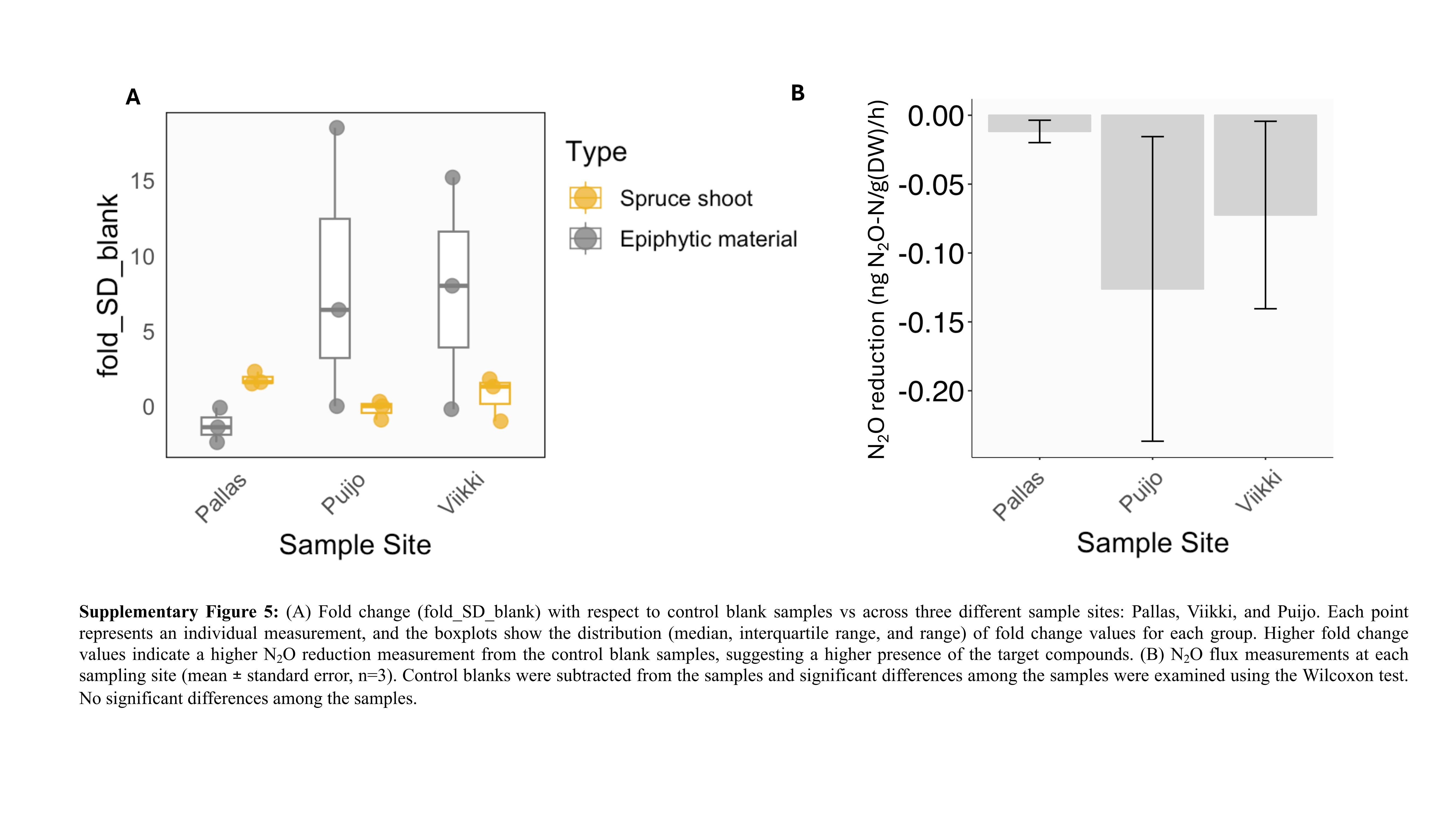

Supplement: FigS5_ycaf196 [file figs5_ycaf196.jpeg]
