# Supplementary material for: Microorganisms in the phyllosphere of Norway spruce controlling nitrous oxide dynamics
Source: ISME Commun. 2025 Nov 3;5(1):ycaf196. doi: 10.1093/ismeco/ycaf196 (PMC12642870; doi:10.1093/ismeco/ycaf196)
Supplement: Supplementary_Table_1_ycaf196 [file supplementary_table_1_ycaf196.docx]

**Supplementary Table S1.** Anions concentrations analyzed with ion chromatography from the epiphytic spruce shoots.

| **Sample Id** | **Location** | **Nitrite (µg  / g (WW))** | **Nitrate (µg / g (WW))** | **Chlorine (µg / g (WW))** |
| --- | --- | --- | --- | --- |
| KR 1 | Pallas | 17.15 | 4.38 | 9.04 |
| KR 2 | Pallas | 6.91 | 2.5 | 7.77 |
| KR 5 | Pallas | 0 | 0.67 | 0.16 |
| Vi 1 | Viikki | 0 | 74.44 | 0 |
| Vi 2 | Viikki | 0 | 8.66 | 6 |
| Vi 3 | Viikki | 1.92 | 1.68 | 3.55 |
| Pu 1 | Puijo | 16.06 | 4.44 | 7.77 |
| Pu 2 | Puijo | 4.35 | 270 | 40 |
| Pu 3 | Puijo | 68.56 | 11.25 | 23.75 |
